# Pheromone of grouped female mice impairs genome stability in male mice through stress-mediated pathways

**DOI:** 10.1038/s41598-023-44647-w

**Published:** 2023-10-17

**Authors:** Timofey S. Glinin, Marina V. Petrova, Veronika Shcherbinina, Anastasia N. Shubina, Anna V. Dukelskaya, Polina V. Starshova, Victoria Mamontova, Alexandra Burnusuz, Alena O. Godunova, Alexander V. Romashchenko, Mikhail P. Moshkin, Philipp Khaitovich, Eugene V. Daev

**Affiliations:** 1 Department of Genetics and Biotechnology, Saint-Petersburg State University, Universitetskaya Emb., 7–9, Saint Petersburg, Russia 199034; 2 Open Longevity, 15260 Ventura Blvd, STE 2230, Sherman Oaks, CA 91403 USA; 3 Endocrine Neoplasia Laboratory, Department of Surgery, University of California, San Francisco, CA 94143 USA; 4 Center of Molecular and Cellular Biology, Skolkovo Institute of Science and Technology, Bolshoy Blv. 30, Moscow, Russia 121205; 5 Laboratory of Higher Nervous Activity Genetics, Pavlov Institute of Physiology, Russian Academy of Sciences, Makarova Emb. 6, Saint Petersburg, Russia 199034; 6 Mildred Scheel Early Career Center for Cancer Research (Mildred-Scheel-Nachwuchszentrum, MSNZ), University Hospital Würzburg, Josef-Schneider Str. 2, 97080 Würzburg, Germany; 7 Department of Biochemistry and Molecular Biology, Biocenter of the University of Würzburg, Am Hubland, 97074 Würzburg, Germany; 8 The Federal Research Center Institute of Cytology and Genetics, SB RAS, Academician Lavrentiev Avenue, 10, Novosibirsk, Russia 630090; 9 International Tomography Center, Institutskaya St., 3A, Novosibirsk, Russia 630090; 10 Federal Research Centre of Biological Systems and Agrotechnologies, RAS, St. January 9, 29, Orenburg, Russia 460000; 11 Center for Neurobiology and Brain Restoration, Skolkovo Institute of Science and Technology, 3 Nobelya St., Moscow, Russia 121205

**Keywords:** Genomic instability, Physiology, Ecological genetics, Olfactory system

## Abstract

Population density is known to affect the health and survival of many species, and is especially important for social animals. In mice, living in crowded conditions results in the disruption of social interactions, chronic stress, and immune and reproductive suppression; however, the underlying mechanisms remain unclear. Here, we investigated the role of chemosignals in the regulation of mouse physiology and behavior in response to social crowding. The pheromone 2,5-dimethylpyrazine (2,5-DMP), which is released by female mice in crowded conditions, induced aversion, glucocorticoid elevation and, when chronic, resulted in reproductive and immune suppression. 2,5-DMP olfaction induced genome destabilization in bone marrow cells in a stress-dependent manner, providing a plausible mechanism for crowding-induced immune dysfunction. Interestingly, the genome-destabilizing effect of 2,5-DMP was comparable to a potent mouse stressor (immobilization), and both stressors led to correlated expression changes in genes regulating cellular stress response. Thus, our findings demonstrate that, in mice, the health effects of crowding may be explained at least in part by chemosignals and also propose a significant role of stress and genome destabilization in the emergence of crowding effects.

## Introduction

High population density is a potent environmental factor that affects the health and survival of mammals. It threatens population welfare through increased competition for resources, predator vulnerability, risk of infectious disease spread^1–3^, and in many populations is associated with an increased mortality rate^4–6^. Living in crowded conditions has also direct effects on individuals’ health, causing immune and reproductive suppression^1,7,8^, as well as increased risk of cardiovascular^9^ and psychological disorders^10^. The direct effects of crowding may be mediated, at least in part, by chronic stress reaction, which is supported by the fact that crowding conditions are often associated with physiological alterations in the regulation of the hypothalamic–pituitary–adrenal (HPA) axis and elevated baseline glucocorticoid (GC) levels^11^. At the molecular level, GCs operate as signaling molecules that affect transcriptional regulation, providing multiple metabolic and immune suppressive effects. In addition, GCs have been shown to elicit anti-inflammatory effects through the induction of damages to nuclear material within an immune cell and alter its capacity to repair DNA damage^12^, thus linking multiorgan HPA axis regulation to changes in metabolic and immune function within cells. There are several potential mechanisms that may elicit stress and multiple health outcomes in high density populations, including increased social conflicts (e.g., intensification of the struggle for domination), lack of resources (e.g., territory and food) and chemical signals produced by other conspecifics in the environment^13^. In contrast to behavioral and ecological problems in high-density populations, much less attention has been paid to investigations into chemosignals and their impact on animal physiology and health.

Olfactory chemosignals, or pheromones, emitted by individuals in crowded conditions are described in several species across the animal kingdom; their effects are primarily developmental delay and reproduction suppression^13,14^. In the nematode *Caenorhabditis elegans,* the “crowding” pheromone facilitates the arrest of nematode development before the onset of sexual maturity, increases the frequency of specialized “dauer” larvae formation and prevents its recovery, even in case of availability of food^14^. In mammals, the only pheromone described to date that is specific to a crowded environment is 2,5-dimethylpyrazine (2,5-DMP), isolated from the urine of grouped female mice^13^. It is a volatile cue, detected by several mouse chemoreceptive organs: main olfactory epithelium (MOE), vomeronasal organ (VNO) and Grueneberg ganglion (GG)^15,16^. The effects of 2,5-DMP are commonly described as reproduction suppression, including puberty delay, decreased pregnancy rates, reduced litter viability and smaller size^13,17,18^. In this study, we proposed that 2,5-DMP has a broader effect on recipient mice, contributing to crowding stress development and its physiological and molecular consequences. This hypothesis was supported by the facts that (1) 2,5-DMP is detected by GG^16^, an olfactory subsystem responsible for alarm pheromones (chemosignals produced by threatened conspecifics) and kairomone (predator scent) detection, and triggers fear and stress reactions. (2) 2,5-DMP has a structural similarity with pyrazine-containing kairomones of mouse predators (e.g., wolf), which elicit avoidance and fear reactions in mice^19^. (3) 2,5-DMP production in mice is under adrenal control^13^, indicating a stress-related regulation of its production. Thereby, we aimed to investigate behavioral and physiological effects of 2,5-DMP, to study its potential role in crowding stress development and to reveal the underlying molecular mechanisms.

We have demonstrated that 2,5-DMP induces aversive behavior and triggers GC elevation in male mice, indicating a stress reaction. Chronic exposure to 2,5-DMP resulted in a significant decrease in immune and reproductive organ weight. In order to identify molecular mechanisms that connect 2,5-DMP-induced stress and glucocorticoid dysregulation to downstream health outcomes, we studied genome stability and transcriptome changes in response to 2,5-DMP exposure. We revealed that 2,5-DMP induced stress-related DNA damages and chromosome aberrations in bone marrow cells. The genome destabilizing effect of 2,5-DMP was mediated by olfactory pathways and stress hormones and completely disappeared after chemical olfactory epithelium inactivation or pharmacological GC and catecholamine blockade. Transcriptomic analysis of bone marrow cells after 2,5-DMP exposure revealed a trend toward changes in the expression of genes involved in the unfolded protein response (UPR) and endoplasmic reticulum (ER) stress, and strongly resembled gene expression changes after restraint stress.

## Results

### 2,5-DMP induces aversion, physiological stress, and immune and reproductive suppression

**Figure 1:**
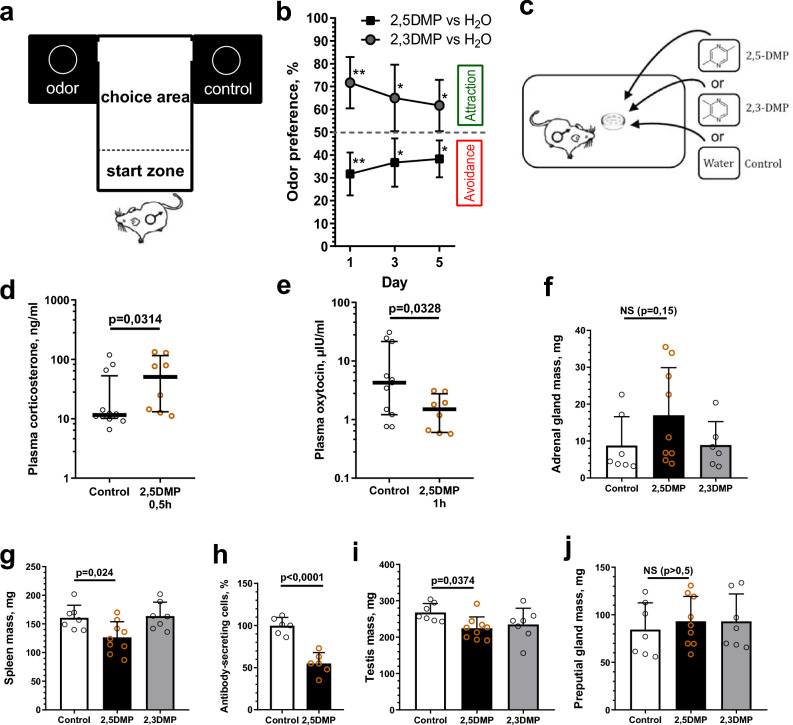
Exposure to 2,5-DMP caused aversion, physiological stress, reproductive and immune suppression in male mice. (**a**) T-maze was used to test the response of mice to odors (2,5-DMP, 2,3-DMP or control H_2_O). (**b**) Percentage of mice odor choices (2,5-DMP vs control—black dots, 2,3-DMP vs control—gray dots) in the two outer sectors of T-maze. Percentage of odor choices more than 50%, significantly different from water, was assumed as “Attractiveness”. Percentage of odor choices less than 50%, significantly different from water, was assumed as “Avoidance”. (n = 10 per group, 6 tests per mouse per day). Wilcoxon signed-rank paired test, *P value ≤ 0.05, **P value ≤ 0.01, mean ± s.d. (**c**) Mice were exposed to the set of odors. For pheromone exposure certain solution (2,5-DMP, 2,3-DMP or control H_2_O) was applied on the filter paper balls in the perforated plastic capsules, which were placed on the male mouse cell grid. (**d**) Plasma corticosterone levels were quantified in mice using enzyme immunoassay after exposure to 2,5-DMP for 0.5 h. (n = 12/8 respectively). (**e**) Plasma oxytocin levels were quantified in mice using enzyme immunoassay after exposure to 2,5-DMP for 1 h. (n = 11/8 respectively). Log10 scales, median with interquartile range and Mann–Whitney test were used for hormonal measurements (**d,e**). (**f,g**) Mean weight changes of mouse adrenal glands (**f**) and spleen (**g**) after 30 days exposure to 2,5-DMP (black column), 2,3-DMP (gray column) or control (white column). (n = 9/7/7 respectively). ANOVA with post hoc Dunnet multiple comparison test. (**h**) Antibody-secreting cells (ASC) in the mouse spleen in response to immunization were quantified using the after 24 h of 2,5-DMP (black column) relative to control (white column) exposure. (n = 6 per group). Unpaired t-test. (**i,j**) Mean weight changes of testis (**i**) and preputial glands (**j**) of male mice after 30 days exposure to 2,5-DMP (black column), 2,3-DMP (gray column) or control (white column). (n = 9/7/7 respectively). ANOVA with Dunnett’s multiple comparison post hoc test. Organ weight data **(f,g,i,j)** were obtained from the CD1 strain, whereas all other experiments utilized the CBA strain. Data **(f,g,h,i,j)** represent mean ± s.d.

Living in crowded conditions is known to induce behavioral and physiological alterations such as social avoidance, increased anxiety^20^, elevated levels of stress hormones, and immune and reproductive suppression^7,8,11^. We hypothesized that, in mice, these effects are mediated, at least in part, by olfactory stimuli produced by crowded conspecifics. Thereby, we studied the effects of the grouped female mice pheromone 2,5-DMP on the behavior and physiology of recipient mice. The aversive/attractive properties of 2,5-DMP were investigated in the T-maze odor preference test, where adult male mice had to choose to approach either 2,5-DMP or water (Fig. 1a). 0.01% 2,5-DMP water solution^21^ was applied on filter paper balls and visually hidden in the “arms” of the maze. In order to compare the effects of 2,5-DMP with structurally similar pyrazine analogs, we used 2,3-DMP (not detected in mouse urine) as a control. The test was repeated three times, on the 1st, 3rd and 5th days. Mice preferred 2,5-DMP significantly less frequently than water, indicating “avoidance” of the stimulus (Fig. 1b). The aversive properties of 2,5-DMP were more pronounced on the 1st day of experiment than on the 3rd and 5th days, indicating a habituation effect. Conversely, 2,3-DMP was approached by mice significantly more often than water, which indicates “attraction” toward the cue (Fig. 1b), which is consistent with the data that pyrazine-containing volatiles are able to activate olfactory neurons and induce behavioral responses in mice, depending on the number and the position of the methyl groups^19^.

To determine whether 2,5-DMP can elicit physiological stress, we measured circulating levels of corticosterone in male mice 0.5 h after the beginning of pheromone exposure (Fig. 1c). ELISA demonstrated that 2,5-DMP exposure led to a significant increase (5 times) in corticosterone plasma levels (Fig. 1d). Another hormone regulating physiological stress is oxytocin, which, on the contrary, suppresses glucocorticoid secretion and downregulates the stress response^22^. 1 h after 2,5-DMP exposure, a significant decrease in the oxytocin level was observed (Fig. 1e), which maybe an extra stress exacerbating factor. A common effect of chronic stress, including stress caused by prolonged crowding, is adrenal hypertrophy^23^. However, we did not reveal an increase in adrenal gland weight after prolonged (30 days) 2,5-DMP exposure (Fig. 1f), which may demonstrate a possible adaptation of the hypothalamic–pituitary–adrenal (HPA) axis to chronic pheromone treatment or indicate that pheromone-induced stress is milder than stress promoted by crowding. Thus, it was shown that transitory 2,5-DMP exposure results in a systemic stress response through HPA axis induction, which may be further enhanced by oxytocin level downregulation.

A well-studied effect of an increase in the serum GC concentration is immune and reproductive suppression^24,25^. Since 2,5-DMP exposure leads to elevated GCs plasma levels, we studied whether it could affect immune and reproductive functions. Prolonged (30 days) exposure of male mice to 2,5-DMP resulted in a 27% decrease in spleen mass (Fig. 1g). This effect was not observed after treatment with the structurally similar pyrazine analog 2,3-DMP (not detected in mouse urine), which we used as a control. The body weight of the mice did not differ between the groups (Supplementary Fig. 1). We also revealed that, after 2,5-DMP exposure, mouse splenocytes demonstrated a reduced level of antibody-secreting cells (ASC) in response to immunization, indicating an impaired humoral response (Fig. 1h). In addition, 30 days exposure of male mice to 2,5-DMP (but not 2,3-DMP) resulted in 21% decrease of testis mass (Fig. 1i). However, 2,5-DMP exposure did not affect the weight of preputial glands (Fig. 1j), which are involved in male reproduction and pheromone production and may be considered as indicators of androgenic activity^23^. This indicates that the testis mass decrease may have occurred due to mechanisms different from androgen downregulation. Thus, it was shown that prolonged 2,5-DMP exposure results in immune and reproductive suppression, such as organ weight decline and functional pathologies.

### 2,5-DMP induces genome instability in mouse bone marrow cells through stress-mediated pathways

**Figure 2:**
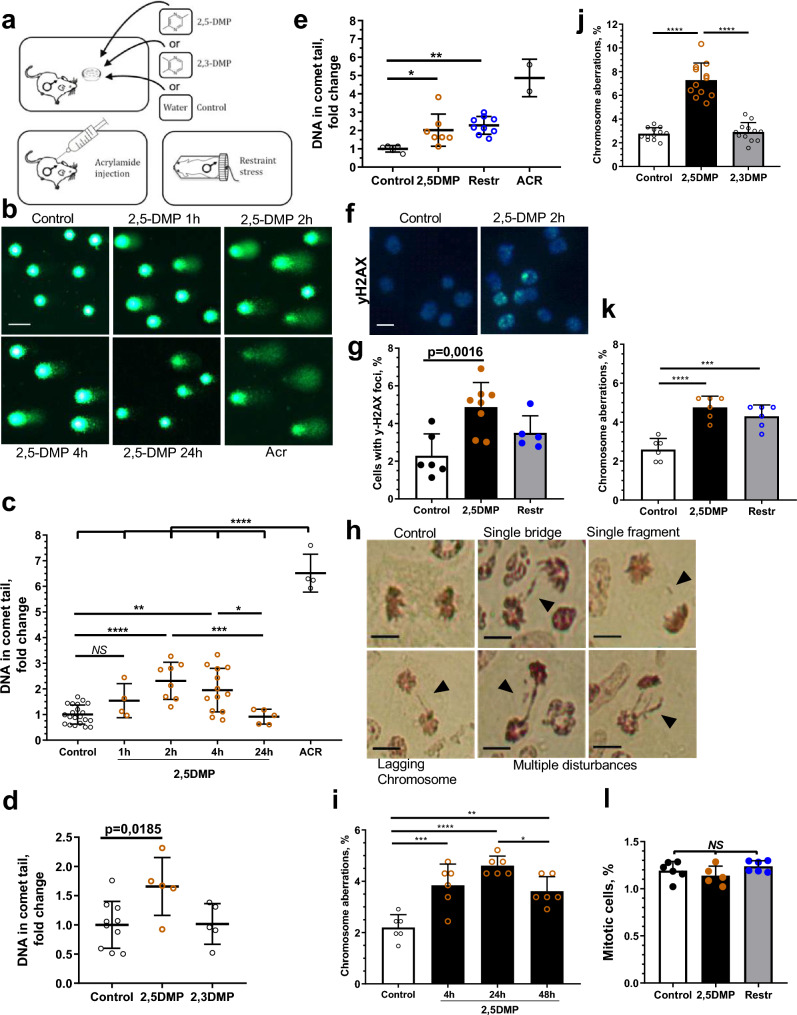
Exposure to 2,5-DMP leads to accumulation of DNA damages and chromosome aberrations in bone marrow cells of male mice. (**a**) Mice were subjected to 2,5-DMP, 2,3-DMP or control H_2_O olfactory exposure, to injection with acrylamide (ACR) or to 2 h of restraint stress (Restr). (**b–e**) Alkaline comet assay of mice bone marrow cells after 2,5-DMP exposure. DNA fragmentation was assessed by the proportion of DNA detected in the tail region. At least 300 comets per mouse were analyzed. Representative images (scale bar, 50 μm) (**b**) and quantitative analyses are shown (**c–e**). Percent of DNA in comet tail relative to control, after different durations of 2,5-DMP exposure (**c**) and 2 h of 2,5-DMP exposure, compared to 2 h of 2,3-DMP (**d**) and 2 h of Restr (**e**). (**c**, n = 21/4/8/13/5/4 respectively, **d**, n = 10/5/5 respectively, **e**, n = 5/9/7/2 respectively). (**f,g**) Bone marrow cells were immunostained for γ-H2AX after 2,5-DMP exposure and Restr. γ-H2AX foci (green); nuclei were stained with DAPI (blue). Scale bar, 10 μm. At least 300 cells per mouse were analyzed. Representative images (**f**) and quantitative analyses are shown (**g**), n = 6/8/5 respectively. (**h–j**) Chromosome aberrations (CA) in dividing bone marrow cells were assessed after 2,5-DMP, 2,3-DMP exposure or Restr. At least 200 divisions at the anaphase-telophase stage per animal were analyzed. (**h**) Representative images of CA and normal division in bone marrow cells after 2,5-DMP exposure. Single bridge, single fragment, lagging chromosome and multiple disturbances were considered as CA. Scale bar, 10 μm. Percentage of bone marrow cells with CA after different durations of 2,5-DMP exposure (**i**), after 24 h of 2,5-DMP or 2,3-DMP exposure (**j**), 22 h after 2 h of 2,5-DMP exposure or Restr (**k**) (**i**, n = 6 per group, **j**, n = 12 per group, **k**, n = 6 per group). (**l**) Mitotic indices in bone marrow cells after 2,5-DMP or Restr. (n = 6/5/6 respectively). Represent mean ± s.d. ANOVA with post hoc Tukey’s multiple comparisons test, *P value < 0.05; **P value < 0.01; ***P value < 0.001, ****P value < 0.0001, not significant (NS) = P > 0.05. All experiments were performed on CBA strain.

On the molecular level, one of the possible mechanisms underlying stress-related immune suppression is the disruption of cell genome integrity by inducing DNA damages and/or altering the capacity of cells to repair DNA damage. A potential role in this process is attributed to stress hormones, i.e. glucocorticoids and catecholamines^12,26^. Previously, we reported that olfactory exposure to the stress-inducing pheromone 2,5-DMP may promote the accumulation of chromosome aberrations^27^. Thus, we studied the effects of 2,5-DMP on various genome maintenance parameters in mouse immune cells. Male mice were preexposed to 2,5-DMP (Fig. 2a), after which their bone marrow cells were analyzed using the alkaline comet assay, which reveals single- and double-stranded DNA breaks and AP-sites. We used the standard mutagen acrylamide (ACR) as a positive control and structurally similar pyrazine analog 2,3-DMP as a negative control. 2,5-DMP (but not 2,3-DMP) induced a rapid increase in DNA break formation, which was after 2 h of exposure and dropped to baseline after 24 h (Fig. 2b–d). This finding was recapitulated when we examined the frequency of bone marrow cells with nuclear foci of γH2AX, one of the earliest and most sensitive markers of DSBs. Using immunocytochemistry, we revealed that 2 h of 2,5-DMP exposure resulted in a 2.1-fold increase in bone marrow cells with γH2AX foci, compared to water exposure (Fig. 2f,g).

DNA damages may promote chromosome aberrations. To quantify the occurrence of 2,5-DMP-induced chromosome disturbances, we assessed the frequency of bone marrow cells with abnormalities in the mitotic segregation of chromosomes. Male mice were exposed to 2,5-DMP for multiple time periods, after which we assessed the frequency of bone marrow cells at the ana-telophase stage with one of the following types of aberrations: lagging chromosome, single fragment, single bridge and multiple disturbances (Fig. 2h). Consistent with the accumulation of DNA damage (Fig. 2c,d), 2,5-DMP (but not 2,3-DMP) resulted in a significant increase in chromosome aberrations (Fig. 2i,j, Supplementary Fig. 2a). Chromosome aberrations occurred in general after a longer exposure period then DNA damages, which indicates that chromosome aberrations may be a result of incorrect DNA damage repair. The percentage of dividing cells and the frequency of certain types of chromosome disturbances did not differ between groups (Fig. 2l, Supplementary Fig. 2b). The ability of 2,5-DMP to induce bone marrow chromosome aberrations was not strain-specific and was repeated in various mice strains (Supplementary Fig. 2a). Thus, it was shown that exposure of male mice to 2,5-DMP leads to DNA damage formation in bone marrow cells, accompanied by the subsequent accumulation of chromosome aberrations, indicating compromised genome maintenance.

In addition, we compared 2,5-DMP exposure with restraint stress, another well-established physiological stress model, which has been shown to induce DNA damage accumulation^28^. We revealed that 2 h of restrained stress, performed using a standard protocol^29^, recapitulated the effects of 2,5-DMP exposure to induce DNA damage (Fig. 2e) and chromosome aberration accumulation (Fig. 2k) in the bone marrow cells of male mice; however, unlike 2,5-DMP, restraint stress did not increase the formation of γH2AX foci (Fig. 2g). Taken together, these results demonstrate that olfactory exposure to the grouped female mice pheromone 2,5-DMP induced more pronounced genome destabilization, as compared to restraint stress, which is a potent stress protocol that combines physical and psychosocial stress components.

**Figure 3:**
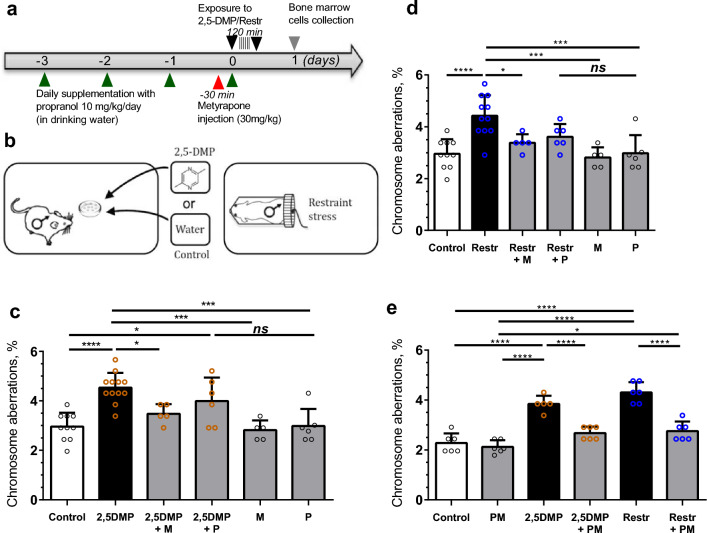
2,5-DMP-induced genome destabilization in male mice bone marrow cells is mediated by stress-related pathways. (**a**) Schematic of the experimental procedure. Adult mice were preexposed to the glucocorticoid blocker metyrapone (M), the β-adrenoblocker propranol or their combination. Propranolol (P) (10 mg/kg/day) was added to drinking water 3 days before and on the day of stress exposure. Metyrapone (30 mg/kg) was injected intraperitoneally 30 min before the beginning of stress exposure. Stress exposure (2,5-DMP exposure or restraint stress) was performed on day 0 and lasted for 2 h. 1 day after the beginning of stress exposure, mice were euthanized, and bone marrow cells were isolated and analyzed by the ana-telophase chromosome aberrations (CA) test. (**b**) Schematic representation of stress exposure. Mice were subjected to 2,5-DMP, control H_2_O olfactory exposure or to restraint stress (Restr). (**c**) The percentage of dividing bone marrow cells with CA isolated from male mice preexposed to P or M, after 2 h of 2,5-DMP olfactory exposure (**c**) or 2 h of Restr (**d**). (**c**, n = 10/11/5/6/5/6 respectively, **d**, n = 10/11/4/6/4/5/6 respectively). (**e**) Percentage of dividing bone marrow cells with CA isolated from mice preexposed to combination of P and M, after 2 h of 2,5-DMP or Restr. (n = 6/6/5/6/6/6 respectively). All the data represent mean ± s.d. ANOVA with Tukey’s multiple comparisons post hoc test. At least 200 divisions at the anaphase-telophase stage per animal were analyzed. *P value < 0.05; ***P value < 0.001, ****P value < 0.0001, not significant (NS) = P > 0.05. All experiments were performed on CBA strain.

On the next step, we investigated the role of stress hormones in 2,5-DMP-induced genome destabilization. Previous studies in vitro and in vivo demonstrated that both GCs and catecholamines are able to induce DNA damage accumulation^26,30^. Thereby, we studied the effect of 2,5-DMP exposure on genome stability in the presence or absence of pharmacological stress hormone blockers: the non-selective β‐adrenoblocker propranolol, and the GC biosynthesis blocker metyrapone. Male mice were pretreated with metyrapone, propranol or their combination, then exposed to 2,5-DMP (or restraint stress), after which their bone marrow cells were extracted and analyzed for chromosome aberrations. The schematic for this study is shown in Fig. 3a,b. The accumulation of chromosome aberrations induced by stressors (2,5-DMP and restraint stress) was slowed down after metyrapone pretreatment (Fig. 3c,d) and totally reduced after simultaneous administration of metyrapone and propranolol (Fig. 3e). Propranolol pretreatment did not result in significant changes, but demonstrated a trend for a reduction in the level of chromosome aberrations after 2,5-DMP and restraint stress (Fig. 3c,d), which may indicate a more substantial role for GCs than catecholamines in 2,5-DMP-induced genome destabilization. Taken together, these data indicate that the genome instability observed in bone marrow cells after 2,5-DMP exposure is stress-dependent and requires the activation of GC and catecholamine pathways.

### Olfactory control for 2,5-DMP-induced genome destabilization

**Figure 4:**
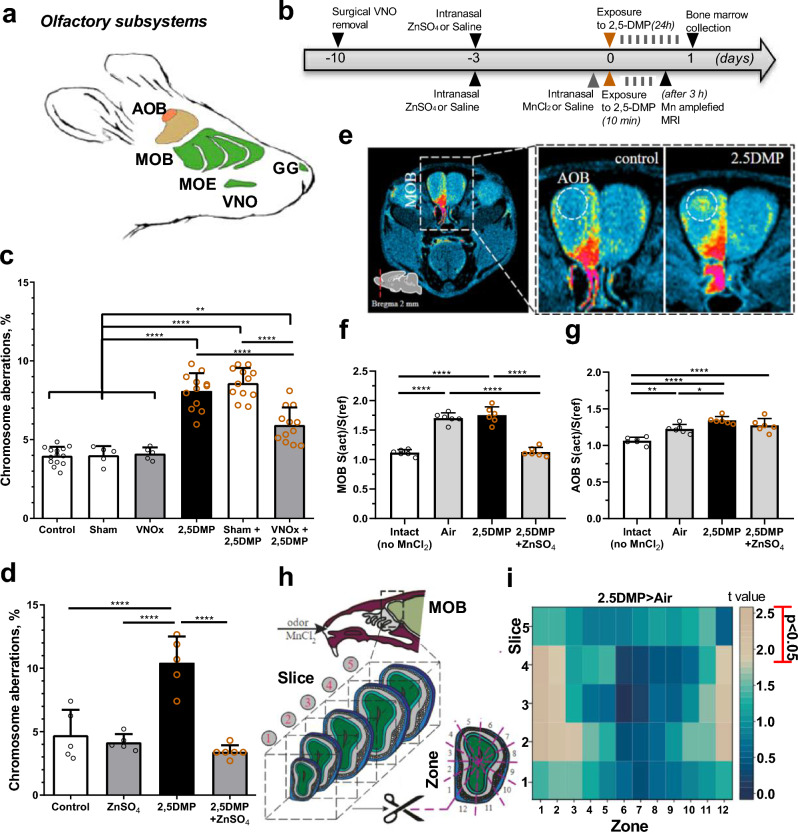
2,5-DMP-induced genome destabilization in male mice is dependent on AOS and MOS. (**a**) Schematic representation of the mouse head and its olfactory subsystems (main olfactory epithelium, MOE; vomeronasal organ, VNO; Grueneberg ganglion, GG; main olfactory bulb, MOB; accessory olfactory bulb, AOB). (**b**) Schematic of the experimental procedures. Mice underwent VNO surgical removal (VNOx) or sham surgery (Sham) 10 days prior to a 24-h olfactory exposure to 2,5-DMP or control H2O on day 0, followed by bone marrow cell isolation. 3 days before exposure, mice underwent intranasal administration of ZnSO_4_ for MOE inactivation. For the Mn-enhanced MRI, mice were administered MnCl_2_ intranasal prior to 10 min exposure to either 2,5-DMP or clean air, and subsequently scanned 3 h post-exposure. (**c,d**) Pheromone-induced chromosome aberrations in bone marrow cells was assessed after VNO removal (**c**) and MOE inactivation (**d**). At least 200 divisions at the anaphase-telophase stage per animal were analyzed. (**e**) T1-weighted MRI scans of intranasally installed manganese distribution through the MOB and AOB (dotted circle) of air- and 2,5-DMP stimulated mice. **(f,g)** Following 2,5-DMP olfactory exposure, the total activity of MOE **(f)** and AOB **(g)** was assessed, with groups pre-treated with ZnSO_4_, exposed to clean air (Air), and without MnCl_2_ administration (Intact). MOB and AOB activity was expressed as normalized T1-weighted MRI reflecting the stimul-induced Mn2 + accumulation. **(h)** The schematic representation of the activity assessment in the olfactory bulbs via Mn-enhanced MRI. Activity was analyzed over five slices and every slice was further divided into 12 distinct zones. **(i)** A heat map displays the significance of changes in the T1-weighted MRI signal across MOB zones in animals exposured to the 2,5-DMP, in comparison to the clean air (Air). Colors on the map represent the t-values obtained for each region through intergroup comparison, gray blocks represent significant difference P < 0.05. **(c,d,f,g)** Data represent mean ± s.d. ANOVA with Tukey’s multiple comparisons post hoc test. *P value < 0.05, **P value < 0.01; ****P value < 0.0001 (**c**, ICR strain, n = 14/5/5/12/12/12 respectively, **d**, CBA strain, n = 5/5/5/6, respectively, **f,g**, CBA strain, n = 6 per group).

2,5-DMP was previously shown to be processed by several mouse olfactory systems, including the accessory olfactory system (AOS)^15,31^, which initiates in the vomeronasal organ (VNO) and is functionally linked to a range of innate behaviors, including fear and stress^32^, and the main olfactory system (MOS), originating in the main olfactory epithelium (MOE)^16,31^ and implicated in wide range odor detection (Fig. 4a). However, which of these olfactory pathways is involved in the control of 2,5-DMP-induced genome destabilization remains unclear. To investigate the role of the VNO in 2,5-DMP effects, we subjected male mice to either surgical removal of the VNO (VNOx) or sham surgery (Sham). Ten days after the procedure mice, were exposed to 2,5-DMP or water for 24 h, after which their bone marrow cells were isolated and analyzed for chromosome aberrations. The schematic for this study is shown in Fig. 4b. Removal of VNO decreased 2,5-DMP-induced accumulation of chromosome aberrations by 1.4 times, as compared to sham and not-operated animals (Fig. 4c). However, the level of chromosome aberrations in the VNOx group after 2,5-DMP exposure was still higher than in the water-treated groups (VNOx, Sham and not-operated). This data indicates that VNO removal reduces 2,5-DMP-induced genome destabilization, but is not sufficient to fully eliminate it, suggesting the involvement the other olfactory pathways.

Next, in order to elucidate the role of the MOS in 2,5-DMP-induced genome destabilization, the MOE was lesioned via intranasal zinc sulfate administration. Mice were administered either zinc sulfate or saline 3 days before the experiment. They were then exposed to either 2,5-DMP or water for a duration of 24 h, after which their bone marrow cells were analyzed for chromosomal aberrations. Our findings showed that lesions in the MOE induced by zinc sulfate completely reduced the 2,5-DMP-induced accumulation of chromosomal aberrations (Fig. 4d).

To further probe the roles of the MOS and VNO in the effects of 2,5-DMP, we assessed the capacity of 2,5-DMP pre-exposure to zinc sulfate to modulate the activity of both the main and accessory olfactory bulb (MOB and AOB) where the MOE and VNO project their axons^16^. Employing manganese-enhanced magnetic resonance imaging (Mn-enhanced MRI) (Fig. 4b,e), we confirmed that 2,5-DMP activates the VNO, as evidenced by the manganese accumulation in AOB neurons in comparison to clean air (Fig. 4e,g). We also demonstrated a complete inactivation of the MOE by zinc sulfate, comparable to the level seen in intact animals that did not receive manganese administration (Fig. 4f). However, intranasal administration of zinc did not result in a complete inactivation of the AOB. Instead, its activity level was intermediate between that of 2,5-DMP and clean air (Fig. 4g).

The capability of zinc sulfate to completely counteract 2,5-DMP-induced genome destabilization, even though VNO pathways are unaffected by zinc sulfate treatment, may suggest an interconnectedness between VNO and MOE pathways in 2,5-DMP processing. This aligns with previous research indicating that odor detection by the MOE is a prerequisite for initiating sampling by the vomeronasal system^33^. These findings suggest that apart from the VNO pathways, the MOS plays a leading role in 2,5-DMP-induced genome instability. The overall activity level of the MOS (Fig. 4f) equally high for 2,5-DMP and air in the accumulation of manganese ions, possibly due to the challenge of ensuring complete absence of extraneous odors (including mouse body odor). However, mapping MOB activity level (Fig. 4h) underscored a significant involvement of ventromedial part in 2,5-DMP processing (zones 1, 2, and 12) across sections 2 to 4 of the olfactory bulbs (Fig. 4i).

### Transcriptomic profiles of mouse bone marrow cells after 2,5-DMP exposure and restraint stress

**Figure 5:**
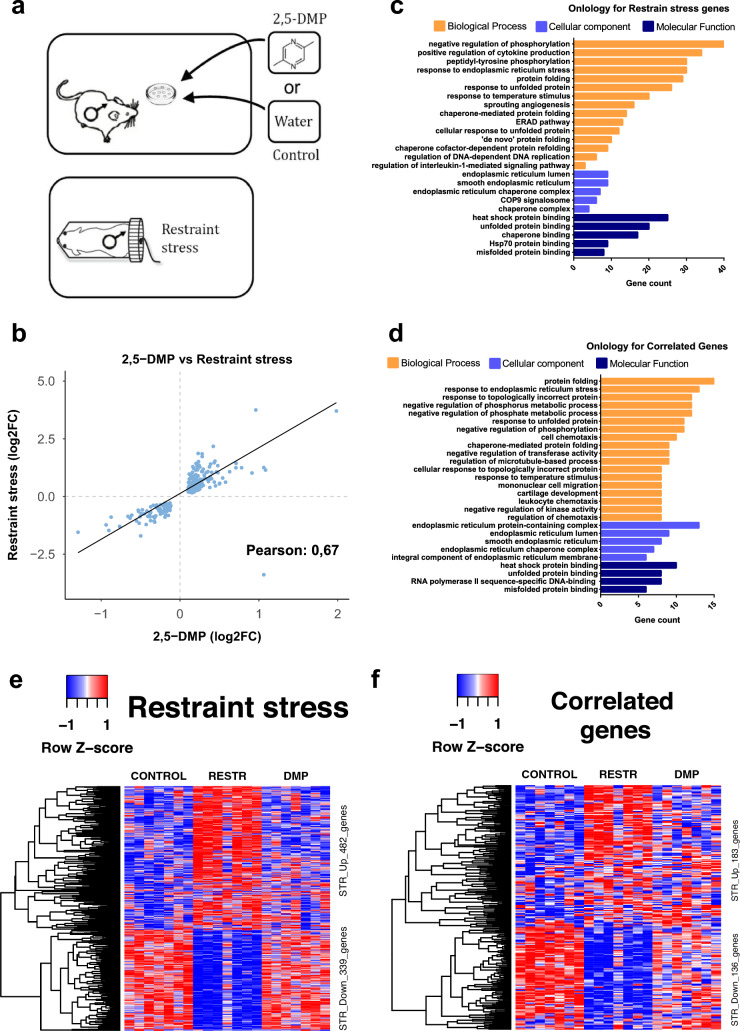
Transcriptomic profiles of the bone marrow cells of male mice after 2,5-DMP exposure and restraint stress (R). (**a**) Schematic of the experimental procedure. Mice were subjected to 2 h of 2,5-DMP olfactory exposure, 2 h of restraint stress (R) or control H_2_O exposure (n = 7/7/7 respectively). (**b**) Scatter plot of 821 differentially expressed genes (DEGs) from RNA-seq data of restraint mice bone marrow tissue, compared to 2,5-DMP exposure (Pearson correlation between the two treatments = 0.67, P < 0.0001). (**c**) The top 25 Gene Ontology terms associated with biological process, cellular component, and molecular function of DEGs after restraint stress. (**d**) The top 24 Gene Ontology associated with biological process, cellular component, and molecular function of genes correlated between restraint stress and 2,5-DMP treatment. (**e,f**) Heatmap of hierarchically clustered z-score normalized log2-transformed mean reads per kilobase per million (RPKM) across all experimental groups based on 821 DEGs after restraint stress (**e**) and on 319 CORR genes (**f**). Hierarchical clustering reveals two clusters that are progressively upregulated (STR-up) or downregulated (STR-down). All data were obtained on CBA strain.

To interrogate the molecular mechanisms underlying stress-induced genome destabilization, we performed RNA sequencing of bone marrow cells isolated from male mice after 2 h of 2,5-DMP exposure and restraint stress (Fig. 5a). RNA-seq analysis revealed 821 differentially expressed genes (DEGs) (FDR < 0.05) after restraint stress (Fig. 5e, Supplementary Fig. 3a) and no significant gene expression changes after 2,5-DMP exposure. However, the expression profiles showed a significant positive linear correlation between restraint stress and 2,5-DMP exposure (Pearson correlation coefficient ρ = 0.67, P < 0.0001), with 319 correlated genes (CORR) (Fig. 5b,f). Gene Ontology (GO) enrichment analysis revealed that the most significantly overrepresented terms in all categories after restraint stress were related to the unfolded protein response (UPR) (Fig. 5c Supplementary Fig. 3b), which was also confirmed for CORR genes (Fig. 5d). Gene expression fold changes correlated positively between stresses for UPR genes (ρ = 0.69, P < 0.0001), with the strongest correlation for ER stress response genes (ρ = 0.68, P < 0.0001), the ERAD pathway (ρ = 0.74, P = 0.004) and Hsp70 protein binding (ρ = 0.92, P = 0.0003) (Supplementary Table 1). Of all the DEGs after restraint stress (but not CORR genes), there was also a robust over-representation of genes implicated in immune-related processes such as cytokine production and the IL-1-mediated signaling pathway. An unsupervised clustering grouped 821 DEGs after restraint stress into up- and downregulated did not reveal more complex expression patterns (Supplementary Fig. 3c,d); among 319 CORR genes only upregulated cluster was revealed (Supplementary Fig. 3e).

In the restraint stress group, the first major cluster of 482 upregulated genes featured predominantly GO terms related to UPR, ER stress and the heat shock response (e.g., Dnajb11, Hsp90b1, Pdia6, Sdf2l1, Pdia4, Dnajb1, Hspa5, Hyou1) (Supplementary Fig. 3c, Supplementary Table 2). In particular, seven out of twelve components of the endoplasmic reticulum multichaperone complex, which function as an ER protein quality control system, had been transcriptionally induced (Dnajb11, Hsp90b1, Pdia6, Sdf2l1, Pdia4, Dnajb1, Hspa5, Hyou1)^34–36^. GO analysis also showed enrichment of T cell activation, cytokine biosynthesis regulation, myeloid cell differentiation and regulation of cell adhesion (genes coding for surface proteins: syndecan-4, ephrin-B3, and Vcam1). We also revealed enrichment in the intrinsic apoptotic signaling pathway (e.g., Hspb1, Ubqln1, Cyp1b1, Stk25, Hsph1, Mcl1, Bcl2) and DNA biosynthesis regulation genes. The second cluster of 339 downregulated genes mainly contained genes involved in the regulation of angiogenesis and endocytic recycling (Supplementary Fig. 3d, Supplementary Table 3). Also, negative regulators of DNA replication were non-significantly overrepresented among cluster 2 repressed genes (Msh3, Tipin, Timeless, Smarcal1, Shld2). KEGG pathways analysis of both clusters identified 13 overrepresented signaling pathways, including protein processing in the endoplasmic reticulum, TNF signaling pathway, estrogen signaling pathway, NF-κB signaling pathway and several cancer pathways (including acute myeloid leukemia) (Supplementary Table 4). In the CORR genes group, the upregulated gene cluster featured predominantly GO terms related to protein folding, response to ER stress and topologically incorrect proteins, UPR and HSPs (Supplementary Fig. 3e, Supplementary Table 5). Taken together, these results indicate that restraint stress induced significant gene expression changes in the bone marrow cells of male mice, which are positively correlated with 2,5-DMP-induced changes. Transcriptomic profiles reveal cellular stress response involving UPR, ER stress and HSP. Based on the current findings, only restraint stress led to statistically significant changes in gene expression within bone marrow cells. Thus, at the transcriptional level, restraint stress might be considered as a more intense stressor compared to pheromone exposure, or a 2-h time point may not be sufficient to observe notable transcriptomic alterations due to 2,5-DMP. On the other hand, since the 2-h duration was chosen based on its sufficiency to induce DNA damage (Fig. 2c,e), this indicates that such damages arise from pre-existing intracellular signaling cascades and do not require additional RNA synthesis.

## Discussion

Our results highlight the important role olfactory cues play in regulating mouse behavior and physiology in crowded living conditions. The pheromone 2,5-DMP produced by grouped female mice was shown to cause behavioral aversion, physiological stress and in the case of prolonged exposure resulted in a decrease in reproductive and immune organ weight in male mice. We also revealed that acute olfactory exposure to 2,5-DMP and restraint stress induced correlated transcriptomic changes in bone marrow cells, including the unfolded protein response (UPR), endoplasmic reticulum (ER) stress and heat shock proteins (HSP). Finally, it was shown that 2,5-DMP induced genome destabilization in bone marrow cells; this process was controlled by olfactory pathways (MOS, AOS) and by stress hormones, thus, providing a plausible mechanism linking the central nervous system, which senses and processes social experiences, to gene regulation in the periphery, where psychosocial stress is translated into poor health outcomes.

Social animals have developed various mechanisms to signal to their conspecifics about population-threatening conditions: the presence of a predator, injury, illnesses and high population density. One of the basic strategies in rodents is emitting socially important chemosignals, demonstrating that the individual is ill, alarmed or stressed^37,38^. These signals induce anxiety and stress in the recipient animal, which allows the animal to cope in the face of challenge and restore homeostasis. However, the chemical nature of these cues remains elusive. The only mouse stress-related pheromone revealed to date is 2-s-butyl-4,5-dihydrothiazole (SBT), which is emitted by mice in threatening conditions and induces glucocorticoid release accompanied with freezing behavior^38^. Notably, this molecule shares structural similarity with a mouse predator (red fox) odor, which is consistent with the hypothesis that prey cues not only signal danger to conspecifics but might also fool the predator by mimicry of its own scent^38,39^. Here, we described a novel mouse stress-related pheromone, 2,5-dimethylpirazine, which is produced by female mice in a crowded environment and causes aversion and corticosterone release in males; its structure is similar to a wolf kairomone^19^. To our knowledge, this is the first demonstration that a pheromone, specific for crowded living conditions, induces aversion and physiological stress, as their function was previously described as reproduction suppression and puberty delay^13,14,17,18^. We speculate that emitting the aversive stress-related pheromone 2,5-DMP has an adaptational role in high-density populations: it may alert conspecifics about threatening conditions, such as a lack of resources (e.g., space and food), and further promote resettlement. When crowding cannot be mitigated, it may lead to detrimental health effects, including retardation of reproduction, immune suppression and chronic stress^7,8,23^. Here, we show that these effects may be mediated, at least in part, by prolonged 2,5-DMP olfactory exposure, which may lead to a decrease in testis and spleen weight. These data coincide with previous 2,5-DMP studies, demonstrating its ability to delay puberty in male mice including growth inhibition of the testes, coagulating and seminal vesicle glands^18^. A reduction in spleen volume was also shown to be a chronic stress marker occurring in GC-dependent manner^40^. Thus, the current study reveals the role of olfactory signals in regulating mouse behavior and physiology in crowded environmental conditions, and may provide insights into population density self-regulation mechanisms.

An important issue in our study is emphasizing the importance of the social modulation of gene regulation, including gene expression levels and genome stability. Here, we provide support for the social chemosignal-induced modulation of genome integrity and gene expression in mouse immune cells, adding to the growing literature on psychosocial stress (e.g., low social status) and gene expression^41,42^, chromatin accessibility^42^ and DNA methylation^43^. In this study, we revealed that 2 h of olfactory exposure of male mice to the grouped female mice pheromone 2,5-DMP led to a significant increase in DNA damage and γH2AX foci, followed by the accumulation of chromosome aberration. Importantly, the level of genome instability after 2,5-DMP exposure was even higher than after another mouse stressor with a more pronounced physical component, i.e. restraint stress. Genome destabilization was stress-dependent and was abrogated by pharmacological GC and catecholamine blockade, which is broadly consistent with the evidence that catecholamines and GCs are able to induce DNA damage accrual both in cell lines and in mice^12,26,28,30^. GCs may impact genome destabilization through intracellular reactive oxygen species (ROS) formation^30^ and catecholamines act through both Gs-PKA and β-arrestin-mediated signaling pathways, synergistically leading to the accumulation of DNA damage^26,28^. Different types of rodent physical stressors, such as restraint stress, exposure to noise, vibration and electric foot shock have previously been shown to induce genome damage^28,29,44^; however, our study provides the first evidence that it may be induced by non-invasive social factor such as a pheromone. The olfactory-based nature of 2,5-DMP was further supported by Mn-enhanced MRI studies, as inactivation of MOS and AOS olfactory systems shown to mediate 2,5-DMP^15,16,31^, and fear odors processing^45^ completely abolished the genome destabilization effects of 2,5-DMP. Also, we noted that although the current study is limited to male mice, we have previously demonstrated that 2,5-DMP induces genome instability in female mice as well^46^, which partly recapitulates the results acquired from males. However, the ability of 2,5-DMP to induce stress in female mice requires further investigation.

Gene expression profiling of bone marrow cells after 2 h of 2,5-DMP exposure and restrained stress revealed correlated transcriptome changes in bone marrow cells with significant activation of the unfolded protein response (UPR), including the ERAD pathway and Hsp70 protein binding. The UPR, which is mainly known to be activated by environmental stressors (e.g., cold, UV light or toxicants)^47,48^, was recently shown to be modulated by physiological stress conditions, such as exposure to predator or predator scent^49^. Although the mechanisms linking the UPR and DNA damage formation are not fully understood, accumulating facts suggest that they are closely intertwined, e.g., increased expression of Hspa5 could facilitate double-strand DNA break repair by activation of the DSB sensing MRN complex^50^. In addition, restraint stress led to robust changes in transcripts related to the immune response and myeloid cell differentiation, which is consistent with the evidence that stress activates bone marrow hematopoiesis, leading to increased output of myeloid lineage immune cells^51^, and upregulates multiple pathways associated with inflammation and cytokine responses^52,53^.

Taken as a whole, our data demonstrate that the pheromone of grouped female mice 2,5-DMP induces aversion and stress in males, and its chronic exposure causes a decrease in immune and reproductive organ weight. On the molecular level, 2,5-DMP and restraint stress led to correlated changes in the UPR and induced significant genome destabilization in bone marrow cells, adding to the growing literature on the genomic effects of physiological stress^26,28,41,42^. However, the majority of previous studies reported genome destabilization after prolonged physiological stress or stress hormone treatment^26,28,29^, while in our study DNA damage accrual occurred after 2 h of pheromonal or restraint stress exposure, which indicates the detrimental physiological consequences of even acute psychological stress and may have broad biomedical implications. In the bone marrow, a source of HSCs and blood cell progenitors, genome destabilization may lead to subsequent mutagenesis in differentiated cells, resulting in either cell death or dysfunction^54^. Moreover, genome destabilization is considered to be a hallmark of aging, a risk factor for tumorigenesis^55^ and neuropsychiatric conditions^56^, and is thought to be one the main causes of age-related hematopoietic stem cell pool exhaustion^54^ leading to subsequent impairment in hemato- and lymphopoiesis. Thus, our study not only reveals the role of social signals in the modulation of crowding effects, but also identifies candidate mechanisms that link the environment to health or fitness-related outcomes.

## Methods

### Animals

Ten-week-old inbred CBA, CD-1 and BALB/c mice, as well as 10-week-old and 21-day-old outbred ICR mail were obtained from the Vivarium of St. Petersburg State University (Russia) and from the Genetic Resources Center of the Federal Research Center Institute of Cytology and Genetics (Novosibirsk, Russia). All the experiments were conducted on male mice which were grouped (5–6 mice per cage). Animals were housed under a regular 12 h dark/light cycle with food and water ad libitum. Experiments were carried out in accordance with the animal protocols approved by the St. Petersburg State University Ethics Committee (#131-03-1) and ARRIVE guidelines. All methods were performed in accordance with the relevant guidelines and regulations. Mice were euthanized by cervical dislocation. For the chromosome aberration assay, bone marrow samples were obtained by flushing the marrow from femur bones using a needle and syringe with a glacial acetic acid–ethanol mixture. For the alkaline comet assay, immunohistochemistry and RNA sequencing, the flushed cells were washed with phosphate buffered saline (PBS, Invitrogen, pH 7.4). All the experiments were conducted on CBA mice, except for the organ weight measurements (CD-1 mice), and VNO removal studies (ICR mice). The chromosome aberration assay to confirm the intrastrain effect of 2,5-DMP was conducted on BALB/c, CBA, CD-1, and ICR mice.

### Animal treatments and used stimuli

The pheromones 2,5-DMP and 2,3-DMP were purchased from Sigma-Aldrich (USA). Stimuli were diluted 1:10,000 (w:v) in dH_2_O^21^. For mouse pheromone exposure, 1.5 ml of the solution was applied onto filter paper balls in perforated plastic capsules (4 cm in diameter), which were placed on the male mice cell grid. The control was dH_2_O exposure. The direct contact of animals with the solutions was excluded. The exposure was carried out for 0.5, 1, 2, 4, 24, or 48 h or 30 days in various experiments. In the case of 48 h or 30 days of exposure, the capsule with 2,5-DMP solution was replaced with a new one every 24 h in order to compensate for active substance evaporation.

To assess the effects of prolonged 2,5-DMP treatment, male mice were exposed to 2,5-DMP, 2,3-DMP or H_2_O for 30 days. At the end of the treatment period, all tested animals were weighed and then sacrificed. The weight of the adrenal and preputial glands, testis and spleen of the male mice was determined.

Restraint stress was performed using a previously described procedure^29^. To induce DNA damages, restraint stress experiments were performed using a 50 ml plastic centrifuge tube with 3-mm holes for air flow and with tail protection.

To assess the involvement of circulating glucocorticoids in the observed pheromone physiological and genetic effects, metyrapone (30 mg per kg, Sigma-Aldrich, USA, dissolved in 150 μl of physiological solution) was intraperitoneally injected 30 min before the beginning of pheromone exposure. To assess the involvement of circulating catecholamines in the observed pheromone physiological and genetic effects, propranolol (10 mg per kg per day, > 99%, Alinda, Russia, dissolved in dH_2_O) was added to mouse drinking water for 3 days before pheromone exposure. The procedure was carried out according to a previously described method^28^.

For olfactory epithelium inactivation, mice received an intranasal administration of 10% ZnSO_4_ (Sigma) 3 days before the beginning of pheromone exposure^57^. For VNO inactivation, 21-day-old mice were subjected to VNO surgical removal according to a previously described method^58^. Ten days after the procedure, the physical state of mice was checked (by weight measurements and behavioral assessments). In the absence of significant differences related to control animals, mice were utilized in the pheromone exposure experiment. Sham operated animals were used as the control.

### T-maze odor preference test

The aversiveness/attractiveness of the odorants (2,5-DMP, 2,3-DMP) for male mice was assessed using the T-maze odor preference test. It was conducted using a T-maze, consisting of an illuminated start box (plastic box opened at the top, 30 × 37 × 30 cm) and two darkened arms (opaque plastic boxes 10 × 10 × 5 cm), where mice could freely move from the start box. For pheromone exposure, filter paper balls moistened with 2,5-DMP, 2,3-DMP (diluted 1:10,000 (w:v) in dH_2_O) or control water were placed in the T-maze arms. A 100 W electric lamp was suspended 1.5 m above the start box. Mice (10 per group) were placed in the middle of the start box individually and had to turn to the left or the right in order to continue through the maze and hide in the goal arm. In the first 2–3 min, mice walked around the start box alternately visiting both arms and generally stopped in one of them (for > 2 min). The latter was considered as a preference for the corresponding stimulus. If a mouse had not reached the end of either arm after 10 min, the experiment was discontinued. The experiment was repeated 6 times for each mouse. Mouse behavior was tested three times (days 1, 3, 5). Each test consisted of 60 preference/avoidance choices, where the corresponding stimulus was tested vs. water. The percentage of odor preference was calculated as (Y/60) × 100%, where Y is number of specific odor preferences from 60 approaches. Statistical evaluation was performed using Wilcoxon signed-rank paired test.

### Chromosome aberration analysis in bone marrow

Bone marrow samples obtained from mouse femurs were fixed with Clark solution (96% ethanol, glacial acetic acid, 3:1) and stored at 1–4 °C. For chromosome aberration analysis, bone marrow cells were stained with aceto-orcein (4% solution) (Sigma-Aldrich, USA), squashed on slides and analyzed using the anaphase-telophase method^59^. The bridges, fragments, lagging chromosomes and multiple disturbances (two or more damage points in a dividing cell) were considered as chromosome aberrations. At least 200 divisions at the anaphase-telophase stage per animal were analyzed. For mitotic index estimation the sum of metaphases, anaphases and telophases per 1000 interphase cells was counted. Cell divisions were analyzed by a single blinded observer.

### Alkaline comet assay

The DNA damage was detected by the alkaline comet assay based on the single-cell gel electrophoresis method (SCGE). Single-cell suspensions with a volume of 150 μl (3 × 10^5^ ml^−1^) were collected and added to 150 μl of 1% low-melting (*t*_m_ < 42 °C, Type VII, Sigma-Aldrich, USA) agarose gel and transferred to a CH-100 solid-state thermostat at 37 °C. The obtained mixture was applied to heated poly-l-lysine (Sigma-Aldrich, USA) microscope slides (37 °C), then covered with 1% universal agarose solution (*t*_m_ < 65 °C). Two slides were prepared for each animal. Then, the mixture was covered with a coverslip (24 × 24 mm) and stored at 4 °C for 10 min. All further operations were conducted in the dark or under a green light. The slides were immersed in lysis buffer at 4 °C for 1 h. After washing with phosphate buffer, the slides were placed into a horizontal electrophoresis chamber (Cleaver Scientific CSLCOM10, UK). Gel electrophoresis was performed for 20 min (1 V/cm) (C.B.S. Scientific, EPS-300 X Mini-Power Supply). The slides were fixed for 5 min in a 70% aqueous solution of ethyl alcohol, dried at room temperature for 1–2 h and stained with 0.1% SYBR Green I (Sigma-Aldrich, USA) solution for 20 min in the dark. The cell nuclei (not less than 300 nuclei per mouse) were imaged using an AxioScope.A1 (Zeiss, Germany) and digital CCD camera (QImaging, QI Click, Canada) with QCapturePro 7 software (Canada). Images were analyzed using TriTek Comet-Score™ Freeware v1.5 software (USA) and the percentage of DNA content in comet tails was calculated. To validate the efficiency of the DNA comet assay, at least one mice was given an intraperitoneal injection of acrylamide (100 mg/kg, dissolved in saline) 8 h prior to the experiment, serving as a positive control. Cell nuclei were analyzed by a single blinded observer.

### Immunofluorescence

Histone H2AX S139 phosphorylation status in mouse bone marrow cells was examined using immunocytochemistry. Cells were fixed for 5 min with 3% paraformaldehyde at room temperature on microscope slides, permeabilized with 0.2% Triton X-100 (Helicon, Russia) for 20 min, and blocked with 1% bovine serum albumin (BSA) (Sigma-Aldrich, USA) in phosphate buffered saline (PBS) for 20 min at 37 °C in a humid incubation box. Slides were then incubated with anti-phospho-H2AX (Ser 139) rabbit antibodies (#9719, Cell Signaling Technology, 1:200) in 1% BSA in PBS, for 45 min at 37 °C in an incubation box. After that, cells were stained with antifade solution containing DAPI (Sigma-Aldrich, USA) and visualized on an Axio Scope.A1 microscope (Carl Zeiss, Germany). Quantitative analyses of the number of cells with foci were carried out in random areas using ImageJ Software (NIH, USA). Not less than 200 cells per mouse were analyzed. Cell nuclei were analyzed by a single blinded observer.

### Hormone measurements

A commercially available enzyme-linked immunosorbent assay (ELISA) kits was used to determine the serum concentration of corticosterone (Mouse Corticosterone ELISA Kit, Cusabio, USA) and oxytocin (Mouse Oxytocin ELISA Kit, Cusabio, USA). All the procedures were performed following the manufacturer’s instructions. A spectrophotometer (Bio-Rad) was used to determine the optical density.

### Cunningham test

The activity of humoral immunity after pheromone exposure was evaluated using a modification of the plaque-forming cell assay^60^. Ten days after the end of olfactory exposure, mice were immunized with sheep erythrocytes (1 × 10^8^). The count of antibody-producing cells (APC) in the spleen was evaluated on day 4 after intraperitoneal immunization. For this purpose, mice were euthanized with diethyl ether, decapitated and the APC number in the spleen of each animal was quantified. Spleen cell suspensions were prepared by teasing the whole spleen through steel wire meshwork into cell culture medium. Cells were washed and made up to 15 ml with a balanced salt solution. Spleen cells were thoroughly mixed with sheep red blood cells and complement solution (to a final concentration 10% each) in a test tube at 37 °C and then spread on microscope slides under sealed cover slips. Slides were sealed with heated paraffin wax and incubated at 37 °C for 1 h (when a maximum number of plaques may be counted). Erythrocytes surrounding antibody-forming cells were coated with the antibody and lysed by complement. Such areas of lysis were observed using an Axio Scope.A1 microscope (Carl Zeiss, Germany). The count of plaque-forming cells per one thousand nucleated cells was calculated^60^.

### Animal preparation and stimuli presentation for Mn-enchanced MRI

Three days prior to 2,5-DMP treatment 24 mice were housed individually and received an intranasal administration of or 10% ZnSO_4_ or saline. To assess the neuronal activation of the MOB and AOB in response to olfactory stimuli, a 10-μl aqueous solution of 10 mM MnCl_2_ (Sigma-Aldrich Co, MO, USA) was rapidly administered into one nostril using a 20-μl micropipette. Mice that did not receive MnCl_2_ administration were in “Intact” group, which was not exposed to any odors*.* After that, the mouse was put into its empty clean cage and were exposed to either clean air, or 50 μl 2,5-DMP (diluted 1:10,000 (w:v) in dH_2_O) in perforated plastic capsules. Exposure to the odor stimulus lasted for 10 min, after which the capsule was removed from the cage. MRI scanning of the OB was performed 3 h after intranasal application of MnCl_2_. The mice were immobilized 3 min before the scanning with a gas mixture (4%) of isoflurane (Isofluran, Baxter Healthcare Corp., USA) and air using an anesthesia machine (The Univentor 400 Anaesthesia Unit, Univentor, Malta). Anesthetized mouse were placed on a heated surface (temperature 30 °C) set in the MR-scanner. Pneumatic sensor for breathing (SA Instruments, Stony Brook, NY, USA) was put under the lower part of animal body.

### Mn-enhanced MRI

The neuronal activity of 2,5-DMP in the mouse MOB and AOB was studied using an ultrahigh-field BioSpec 11.7 T BioSpec 117/16 USR (Bruker, Germany) MR-scanner for Mn-enhanced MRI study. The neuronal activity of olfactory epithelium and VNO was assessed based on the level of the MRI signal in the glomerular layer of the MOB and in the AOB. Accumulation of manganese ions (Mn^2+^) in neurons of MOB and AOB is very reliably correlated with the level of activity of calcium channels of olfactory epithelial cells and VNO^61,62^. The accumulation of manganese ions in OB cells was expressed as the ratio of the tissue MRI signal level to the level of the MRI signal in the reference, which was tube with saline (0.5 ml) placed along the mouse’s head. MRI scanning was performed at 3 h after the exposure to the 0.01% of 2,5-DMP or clean air. This time is enough for odor-induced transport of Mn^2+^ for nasal cavity to the MOB and AOB^62^. Manganese distribution was mapped by T1-weighted MRI by the RARE (Rapid Acquisition with Relaxation Enhancement) method. Parameters of the pulse sequence of the method (TE = 10 ms, TR = 400 ms), image parameters (field of view—2 × 2 cm, matrix—256 × 256 pixel array, thickness of slice—0.5 mm, 100 μm × 100 μm × 0.5 mm voxel dimensions, the distance between the slices—0.5 mm, the number of slices is 5, the orientation of the slices is coronary), the total scan time was 6 min. MRI scans were processed using ImageJ, involving several steps: image alignment, delineation of the mouse brain boundaries, and image resizing. The alignment of brain geometry and dimensions enabled automated comparison of MRI signal levels in the accessory olfactory bulb (AOB) and specific regions of the main olfactory bulb (MOB) across animals. For analysis, the glomerular layer of the olfactory bulbs in each slice were divided into 12 zones. Within these zones, the MRI signal was averaged, followed by various intergroup comparisons to assess changes in neuronal activity in response to olfactory stimuli. Comparison between 2,5-DMP and clean air on the main olfactory bulbs was conducted by constructing a two-dimensional t-map.

### RNA extraction and sequencing

The bone marrow plug was flushed from the tibia and femur using 500 μl of cold PBS into a 1.5 μl microcentrifuge tube and re-suspended. The cell solutions were centrifuged at 1300×*g* for 5 min at 4 °C, and the supernatant was removed. Seven replicates of mouse bone marrow samples for each condition were used for RNA extraction. Tubes with tissues were snap frozen in liquid nitrogen and stored at − 80 °C for later use. Total RNA was isolated with the QIAzol Lysis Reagent following the manufacturer’s instructions (Qiagen, Hilden, Germany). The RNA yields were quantified by a Qubit 2.0 fluorometer (Life Technologies, Carlsbad, CA, USA), and RNA integrity was verified by the 2100 Bioanalyser (Agilent Technologies, Palo Alto, CA, USA) on RNA Nano chips. Total RNA was processed for paired-end deep sequencing on an Illumina HiSeq 4000 platform following the manufacturer’s instructions for the Illumina TruSeq Sample Preparation Kit v2 (Illumina, San Diego, CA, USA). All 21 indexed libraries were 150 nucleotides paired-end, with uniform sequencing depth across the samples and a median depth of 23 million reads (5% and 95% quantiles of 18 and 50 million reads).

### Estimation of gene expression levels

Adapter sequences were trimmed from reads by Cutadapt (parameters -m 75 –trim-n) ^63^. Original sequencing reads or adapter-trimmed reads were mapped to the reference UCSC (mm10) mouse genome using the spliced aligner HISAT2 (parameters -p 10)^64^. Duplicated reads arising from the library preparation procedure were removed by SAMTools^65^. Uniquely mapped reads were counted by HTSeq (parameters -r pos -f bam -s s = no)^66^ using corresponding data from the mouse (mm10) GENCODE VM15 GTF file as the annotation source. Differential expression analysis was performed using the R Bioconductor package DESeq2^67^. A statistically significant set of genes was selected using a Benjamini and Hochberg false discovery rate (FDR) cutoff set to 0.05. To estimate gene expression changes between conditions, a linear model was fitted for each gene using the binary logarithm of RPKM for each sample as the dependent variables and the experimental condition for the same sample as the independent variables (Benjamini and Hochberg FDR-corrected P < 0.05). The conditions were randomly classified as one of three states: 0, 1 or 2. Linear regression was applied to the genes with statistically significant expression changes between the restraint stress group and the control group (FDR < 0.05), and genes without significant changes in the difference between the pheromone 2,5-DMP stress group and the control group (P < 0.05, FDR > 0.05). We defined linear-model genes (or condition-dependent genes) as the ones with an absolute difference of more than 0.1 in the log2-transformed mean RPKM between the pheromone 2,5-DMP stress group and the control group and monotonically increasing or decreasing expression. The Bioconductor packages “topGO” and “clusterProfiler”^68^ were used for Gene Ontology (GO) enrichment analysis. GO term categories were considered significantly overrepresented using the adjusted P < 0.05 cutoff of Fisher’s exact test. The Kyoto Encyclopedia of Genes and Genomes (KEGG) database was used to determine signaling pathways. Unsupervised hierarchical clustering (function “hclust” in R) was used to identify co-expression modules based on 821 genes differentially expressed in the bone marrow of mice under restraint stress. Gene expression levels were calculated as RPKM (reads per kilobase per million mapped reads). To assess concordance between transcriptional responses induced by the stress pheromone 2,5-DMP and restraint stress in mouse bone marrow tissue, the Pearson correlation coefficient of the gene expression fold change was calculated.

### Statistics

Data are presented as mean ± S.D., unless otherwise stated. All the collected material was coded for the unbiased primary data assessment. The statistical analysis began with the decoding of the primary data. The normality of the intragroup distribution was assessed using the Kolmogorov–Smirnov test. All of the analyses were conducted using Graph Pad Prism 7™ software (GraphPad Software, San Diego, USA). In the case of the normal intragroup data distribution, a two-tailed Student’s t-test was applied for single comparisons between two groups, and ANOVA with the Tukey HSD post-hoc test was used for multiple comparisons. In the case of a non-normal intragroup distribution, the Mann–Whitney test was applied for single comparisons between two groups. Summary of statistical evaluation available in Supplementary Table 6.

### Supplementary Information

Supplementary Legends.

Supplementary Table 1.

Supplementary Table 2.

Supplementary Table 3.

Supplementary Table 4.

Supplementary Table 5.

Supplementary Table 6.

Supplementary Figures.

## Data Availability

Further information and requests for resources and supporting data will be fulfilled by the corresponding authors, T.G. and E.D.
