# Supplementary material for: Pheromone of grouped female mice impairs genome stability in male mice through stress-mediated pathways
Source: Sci Rep. 2023 Oct 17;13:17622. doi: 10.1038/s41598-023-44647-w (PMC10582102; doi:10.1038/s41598-023-44647-w)
Supplement: Supplementary file 1 — Supplementary Legends. [file 41598_2023_44647_MOESM1_ESM.docx]

*Supplementary Table 1. Gene Ontology (GO) enrichment analysis of correlation RNA-seq data of restraint and 2,5-DMP exposure mice bone marrow tissue.*

*Supplementary Table 2. Upregulated genes cluster in mouse bone marrow tissue after restraint stress.*

*Supplementary Table 3. Downregulated genes cluster in mouse bone marrow tissue after restraint stress.*

*Supplementary Table 4.*

*KEGG pathways analysis of RNA-seq data of restraint mice bone marrow tissue.*

*Supplementary Table 5.*

*Upregulated genes cluster of Correlated genes in mouse bone marrow tissue after restraint stress and 2,5-DMP exposure.*

*Supplementary Table 6.*

*Statistical values summary. Spreadsheet demonstrates statistical method with indication of t- and f- value for data presented in fig. 1-4*
