# Supplementary material for: Pheromone of grouped female mice impairs genome stability in male mice through stress-mediated pathways
Source: Sci Rep. 2023 Oct 17;13:17622. doi: 10.1038/s41598-023-44647-w (PMC10582102; doi:10.1038/s41598-023-44647-w)
Supplement: Supplementary file 8 — Supplementary Figures. [file 41598_2023_44647_MOESM8_ESM.docx]

***Supplementary FIG1***

***Supplementary Figure 1.*** *Mean weight changes of body mass of male CD-1 mice after 30 days exposure to 2,5-DMP (black column), 2,3-DMP (gray column) or control H_2_O (white column). (n = 7 Control, n = 9 2,5-DMP, n = 7 2,3-DMP). ANOVA with Dunnett’s multiple comparison post hoc test. Not significant (NS) = P > 0.05.*

***Supplementary FIG 2***


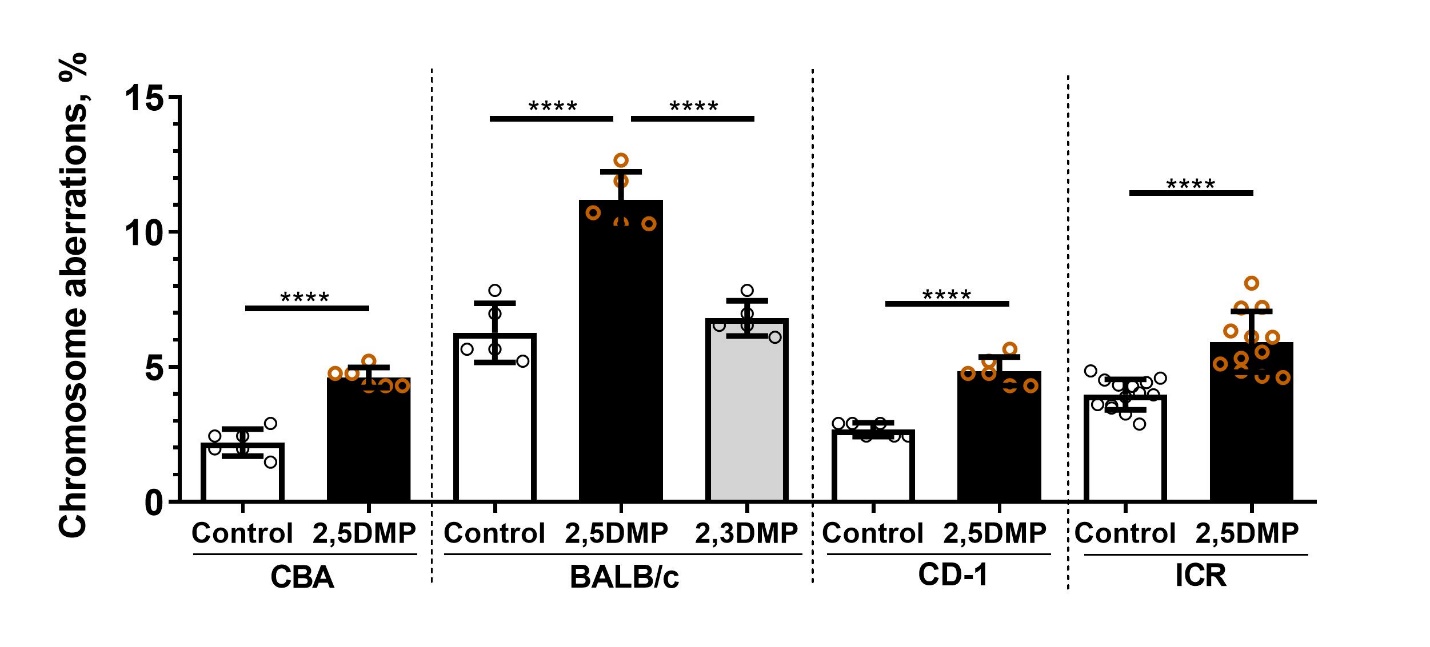


***Supplementary Figure 2a.*** *Percentage of bone marrow cells with chromosome aberrations after 24hs of 2,5-DMP exposure (or control H_2_0 exposure) in CBA, BALB/c, CD-1 and ICR mice. (n=6/6/5/5/5/6/6/14/12 respectively).* For BALB/c additional group of 24h exposure of 2,3-DMP are presented. *For CBA, CD-1, ICR: Mean ± s.d., Student’s t test, **** P value <0,0001 (T-value: 9,469; 8,990; 5,726 respectively).* For *BALB/c ANOVA with post hoc Tukey's multiple comparisons test **** P value <0,0001, (F-value* 39,79)


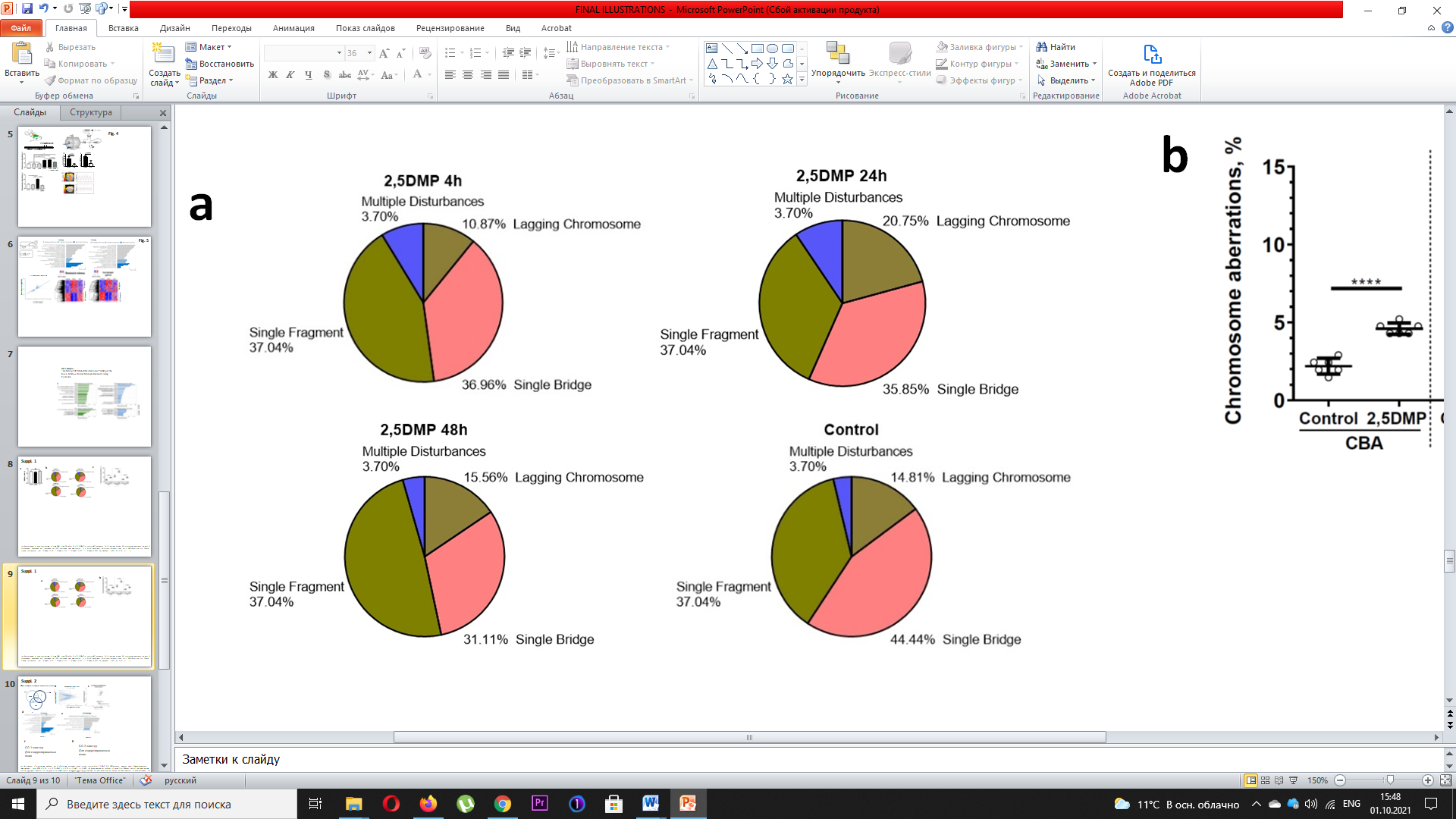


***Supplementary Figure 2b***. Frequency of different types of chromosome disturbances (lagging chromosome, single fragment, single bridge and multiple disturbances) in bone marrow cells of male mice after 4 h, 24 h, 48 h of 2,5-DMP or control H_2_O exposure. (n= 6 per group). Chi-squared test, not significant difference between treatments.

***Supplementary FIG 3***

**MA plot Restraint stress**

**Log2 (Mean Expression)**

**Log2 (Fold Change)**

******

***Supplementary Figure 3a.*** *Minus average plot (MA-plot) of genes from bone marrow RNA-seq data of mice restrained for 2 h, compared to control. The binary logarithm of the mean of normalized counts across all bone marrow samples for each gene is plotted versus the binary logarithm of gene expression fold change. Blue points represent differentially expressed genes (DEG) (FDR < 0,05). Points which are fall out of the y-axis range are plotted as triangles.*

***Supplementary Figure 3b.*** *Heatmap displays the relative gene expression of bone marrow cells of mice subjected to restraint stress (Restr), 2,5-DMP (DMP) and H_2_O (control), that overlap with “Response to unfolded protein” GO term.*

**c**

***Supplementary Figure 3c.*** *The top 25 Gene Ontology terms associated with biological process, cellular component, and molecular function of 482 cluster 1 (upregulated) DEGs after restraint stress.*

**d**

***Supplementary Figure 3d.*** *The top 3 Gene Ontology terms associated with biological process, cellular component, and molecular function 339 cluster 2 (downregulated) DEGs after restraint stress.*

***Supplementary Figure 3e.*** *The top 24 Gene Ontology terms associated with biological process, cellular component, and molecular function of upregulated genes cluster of CORR genes (genes correlated between restraint stress and 2,5-DMP treatment).*
